# Local and Global Protein Interactions Contribute to Residue Entrenchment in Beta-Lactamase TEM-1

**DOI:** 10.3390/antibiotics11050652

**Published:** 2022-05-13

**Authors:** André Birgy, Mélanie Magnan, Claire Amaris Hobson, Matteo Figliuzzi, Karine Panigoni, Cyrielle Codde, Olivier Tenaillon, Hervé Jacquier

**Affiliations:** 1 IAME, UMR 1137, INSERM, Université de Paris Cite, 75014 Paris, France; andre.birgy@aphp.fr (A.B.); melanie.magnan@inserm.fr (M.M.); claire.amaris.hobson@gmail.com (C.A.H.); kpanigoni@gmail.com (K.P.); ccodde@yahoo.com (C.C.); 2 Service de Microbiologie, Hôpital Robert-Debré, AP-HP, 75019 Paris, France; 3 UPMC, Institut de Calcul et de la Simulation, Sorbonne Universités, 75006 Paris, France; matteo.figliuzzi@gmail.com; 4 Computational and Quantitative Biology, UPMC, UMR 7238, Sorbonne Universités, 75006 Paris, France; 5 Service de Bactériologie-Hygiène, Groupe Hospitalier Saint-Louis–Lariboisiére-Fernand Widal, AP-HP, 75010 Paris, France

**Keywords:** entrenchment, protein stability, TEM-1 beta-lactamase, CTX-M-15 beta-lactamase, M182T mutation

## Abstract

Due to their rapid evolution and their impact on healthcare, beta-lactamases, protein degrading beta-lactam antibiotics, are used as generic models of protein evolution. Therefore, we investigated the mutation effects in two distant beta-lactamases, TEM-1 and CTX-M-15. Interestingly, we found a site with a complex pattern of genetic interactions. Mutation G251W in TEM-1 inactivates the protein’s function, just as the reciprocal mutation, W251G, does in CTX-M-15. The phylogenetic analysis revealed that mutation G has been entrenched in TEM-1’s background: while rarely observed throughout the phylogeny, it is essential in TEM-1. Using a rescue experiment, in the TEM-1 G251W mutant, we identified sites that alleviate the deviation from G to W. While few of these mutations could potentially involve local interactions, most of them were found on distant residues in the 3D structure. Many well-known mutations that have an impact on protein stability, such as M182T, were recovered. Our results therefore suggest that entrenchment of an amino acid may rely on diffuse interactions among multiple sites, with a major impact on protein stability.

## 1. Introduction

Beta-lactam antibiotics are among the most prescribed antimicrobial agents, but their efficacy is constantly threatened by beta-lactamases, enzymes that hydrolyze beta-lactams. To date, several thousands of beta-lactamases have been described. These enzymes undergo two evolution speeds: a slow evolution for millions of years [1], and a rapid evolution selected under antimicrobial selective pressure during these last decades [2]. Hence, understanding the constraints that shape protein evolution is not only a matter of basic science but also of public health. 

The chemical and physical properties of a protein, defined by the amino acids’ interactions in three-dimensional space, determine its function. Most constraints acting on these residues remain unknown, and their impact on the protein’s functions is complex. An analysis comparing homologous protein sequences enables us to better identify the constraints acting on the protein’s residues. A conserved residue over a long evolutionary time period signs a strong constraint. However, it is now acknowledged that evolution depends on interactions between residues and that the independence of each site cannot be affirmed [3,4]. This observation therefore leads to “epistasis”, i.e., interactions between mutations: the effect of two mutations combined may be different from that of the sum of the mutations taken separately. This implies a context-dependency effect of mutations, relying on the background surrounding a mutation.

An extreme case of epistasis is referred to as a compensated pathogenic deviation (CPD) [5]. Here, a pathogenic amino acid variant in one specie is observed fixed in the orthologous protein (sharing the same function specificities) of another specie. As an example, in BBS4 gene, H165 mutation is highly conserved in different species, whereas N165 is found in humans. Interestingly, N165H mutation is associated with ciliopathies in humans, whereas this mutation should be expected as neutral [6]. Hence, this mutation has probably been compensated by coevolving residues, and, therefore, it might be deleterious in the absence of associated substitutions. This hypothesis defines “entrenchment” and suggests that proteins tend to balance the presence of an amino acid at a given position through replacements at other positions [7].

To explore the context-dependency effect of mutations, we confronted two libraries of mutants of TEM-1 and CTX-M-15 beta-lactamases. These two enzymes are of medical concern because of their epidemiological success, but also of evolutionary interest because of their high allelic diversity. Interestingly, they are phylogenetically distant, with only 30% identity; however, they have quasi-superposable three-dimensional structures.

Here, we identified a residue entrenched in TEM-1. Subsequent experiments allowed us to hypothesize that amino acid entrenchment may rely on various interactions among multiple sites, leading to a major impact on protein stability.

## 2. Results

### 2.1. Distribution of Single Mutant MICs of Two Beta-Lactamases Reveals a Single Opposite Effect

In search of context-dependency, we first screened the effects of mutations in the two orthologous beta-lactamases, TEM-1 and CTX-M-15. For TEM-1, the data were collected from Firnberg et al.’s study [8]. The authors determined the MIC of amoxicillin for 5041 single missense TEM-1 mutants. For CTX-M-15, we produced 404 single mutants, using random mutagenesis, and determined the MIC of amoxicillin (see methods).

**Figure 1:**
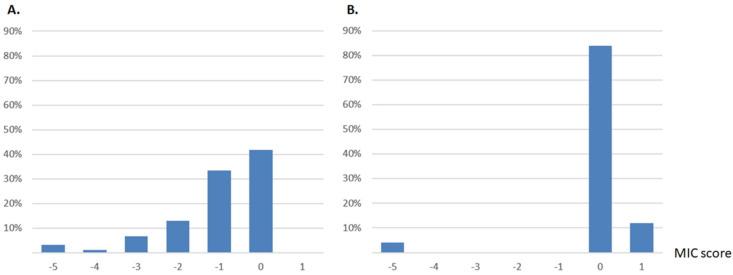
(**A**) Effects of mutations on MIC score when changing TEM-1 residue with that of CTX-M-15 (*n* = 236, dataset from Firnberg et al. [8]). (**B**) Effects of mutations on MIC score when changing CTX-M-15 residue with that of TEM-1 (*n* = 25). MIC score corresponds to log_2_(MIC_mutant_/MIC_WT_). For each collection, one single mutant harbored an inactivating mutation (MIC score = −5): G251W in TEM-1, and W251G in CTX-M-15.

We searched for mutations with a marked context-dependency. We focused on mutants of TEM-1 for which the mutated amino acid at a given site corresponds to the amino acid naturally found at the same position in CTX-M-15, and vice versa. We found 236 single mutants of TEM-1 (Firnberg’s study [8]) and 25 single mutants of CTX-M-15 (this study) that corresponded to this criterion (Figure 1). Among all of these mutations, we spotted one site with a strong context-dependency signature. 

On residue 251, the natural residue is a glycine (G) in TEM-1, and a tryptophan (W) in CTX-M-15. A G251W mutation in TEM-1, and a W251G mutation in CTX-M-15 both lead to loss of function (Figure 1). 

### 2.2. Phylogeny and Local Environment of Site 251 Highlights a Potential Entrenched Site

We used an alignment of 157 class A beta-lactamases to better understand the tolerance of site 251 to mutations, and its conservation through the phylogeny of beta-lactamases [9]. 

**Figure 2:**
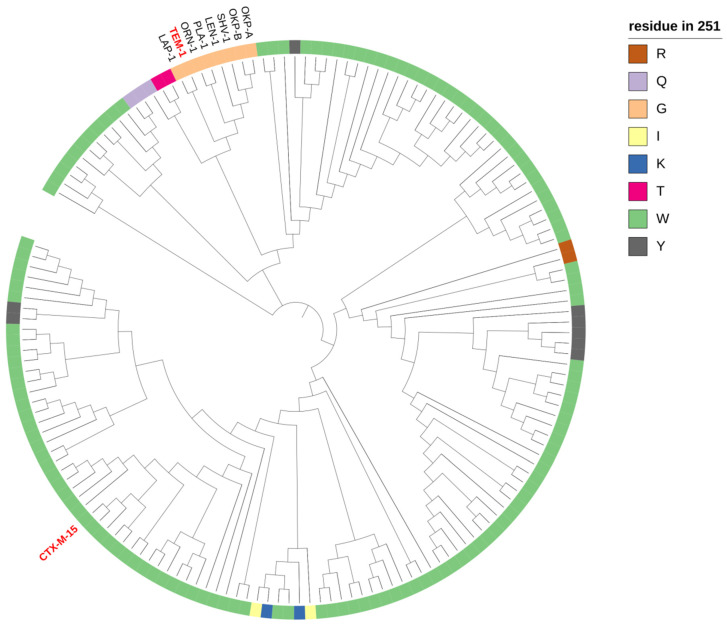
Phylogenetic tree of 157 class A beta-lactamases [9]. The different colors indicate the diversity at position 251. Beta-lactamases with a G251 are labeled in black, and TEM-1 and CTX-M-15 beta-lactamases are labeled in red.

Most beta-lactamases, such as CTX-M-15, harbor a tryptophan at this site (*n* = 130/157), a tyrosine (*n* = 8/157), a glycine (*n* = 8/157), a glutamine (3/157), a lysine (*n* = 2/157), an arginine (*n* = 1/157), a threonine (*n* = 1/157), or an isoleucine (*n* = 1/157) (Figure 2). Beta-lactamases containing G251 belong to a monophyletic group of eight enzymes: TEM-1, OKP-A, PLA-1, OKP-B, LEN-1, SHV-1, ORN-1, and LAP-1. 

By using a simple independent model, we can predict the effect of a mutation at a given position by looking at the frequency of this amino acid at this same position. In our alignment of 157 class A beta-lactamases, W251 was frequent, and G251 was quite rare. The deleterious effect of W251G in CTX-M-15 is, therefore, not surprising. Conversely, the deleterious effect of G251W in TEM-1 could be counterintuitive. This observation suggests not only high epistatic interactions with G251 in TEM-1, but also that G251 could have evolved as a potential entrenched site.

### 2.3. Analysis of Conserved Sites Suggests Local Co-Evolution

To go further, by targeting other amino acids exclusively present in the monophyletic group of eight enzymes that harbor G251 and are close to TEM-1, we searched for sites that could have co-evolved with site 251. 

**Figure 3:**
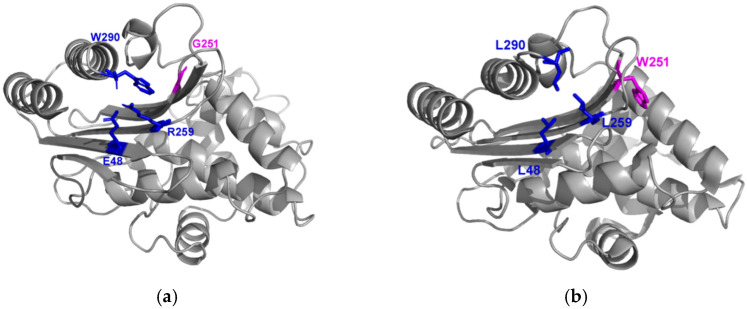
(**a**) Three-dimensional structure of TEM-1 (pdb entry 1BTL). The site G251 is shown in pink, and the 3 residues exclusively associated with G251 (E48, R259, and W290) are indicated in blue. (**b**) Three-dimensional structure of CTX-M-15 (pdb entry 4HBT). The site W251 is shown in pink, and the 3 residues associated with W251 (L48, L259, and L290) are indicated in blue.

**Figure 4:**
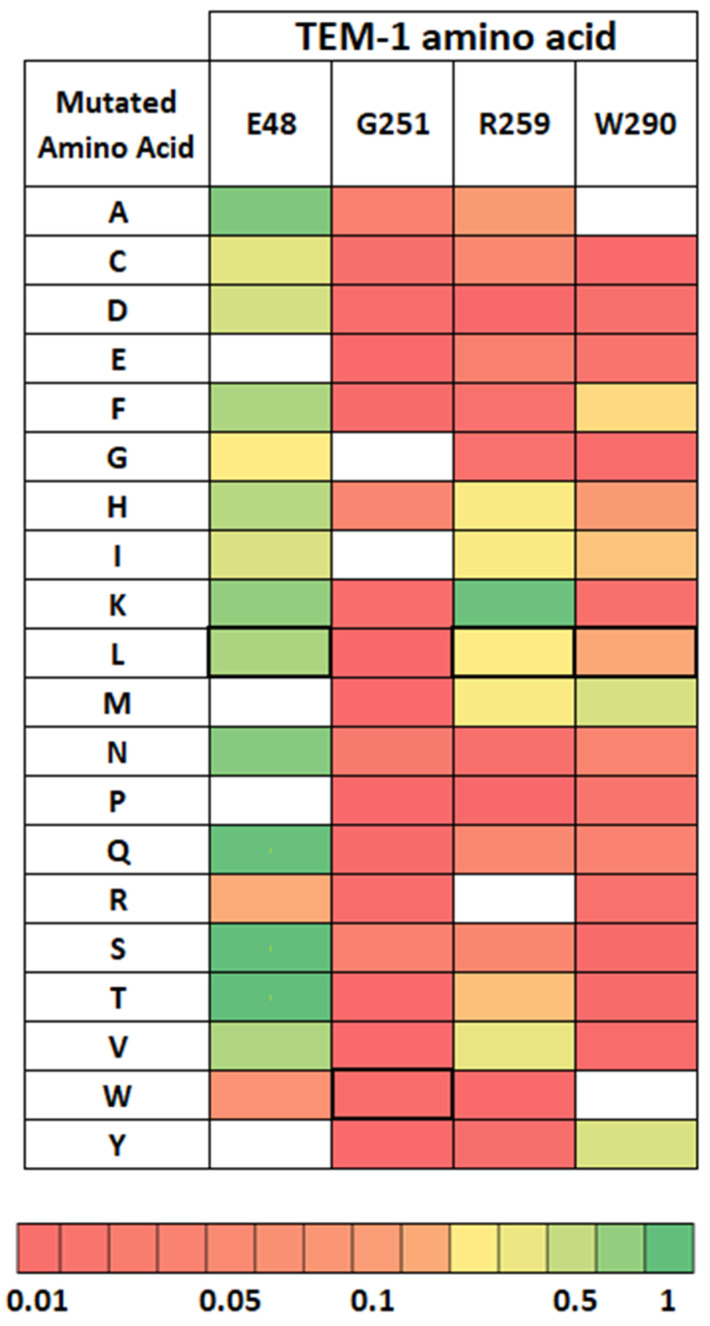
Selective mutational effect of mutations in the 3 amino acids found exclusively in the G251 group (*n* = 8) (E48, R259, and W290). Fitness was obtained by using Firnberg’s data on TEM-1 [8]. A fitness of 1 (green) corresponds to the fitness of the wild-type allele (TEM-1). A fitness of 0 (red) corresponds to a minimum fitness. The color gradient from green to red corresponds to the fitness gradient. The boxed mutations correspond to amino acids present in CTX-M-15.

We found three residues (E48, R259, and W290) that seem to have co-evolved with G251. Interestingly, these three residues are close in the protein (Figure 3 and Supplementary Table S2). This could suggest local epistasis compensating for a deleterious effect of glycine in 251. As observed in Firnberg’s data and suggested by a phylogeny analysis [8,9], these three sites are more tolerant to mutations than G251. However, when replacing the amino acids with the ones of CTX-M-15 at the same site, in these three sites, we observed a moderate decrease in fitness (Figure 4). Hence, this cluster of sites may mutually contribute to the entrenchment of site 251. 

Of note, among the 197 TEM alleles listed in BLDB database [10], none of these sites harbors mutations. This confirms their importance in TEM beta-lactamases.

### 2.4. Distribution of Compensating Mutations in TEM G251W Displays a Various Pattern of Epistatic Interactions

To unravel the sites contributing to the CPD state of glycine at position 251 in TEM-1, we explored the distribution of the compensating mutations in the inactivated mutant G251W after a mutant selection on amoxicillin. We performed a comprehensive mutagenesis by using a pool of mutagenic primers containing a degenerated codon NNS for every site of the protein. To avoid reversion to the wild-type protein, we did not use the mutagenic primers targeting site 251 (see methods).

**Figure 5:**
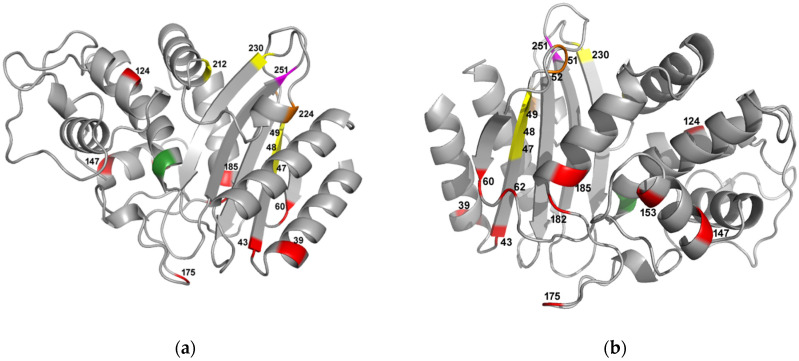
Two opposite faces of the protein (**a**,**b**). Sites with compensating mutations on G251W-TEM variant (pdb entry: 1 BTL) are indicated with different colors. The active site (S70) is indicated in green and site 251 in pink. Mutations with local effects (<13 Å from W251) are indicated in yellow, and those with a global effect are in red. Mutations located <13 Å from W251 but previously described with a global stabilizing effect on the protein are indicated in orange.

After filtering out insertions, deletions, and low-quality sequences, 13 million reliable reads were retained. We defined a cutoff of a 2-fold increase to define mutation as compensatory (see methods). We found 50 compensatory mutations, corresponding to 0.89% (50/5869) of all possible mutations (Supplementary Table S2). These mutations were distributed among 23 different sites, including known compensating hotspots (Figure 5 and Supplementary Materials text).

#### 2.4.1. Global Versus Local Epistasis

Based on the 3D structure of the protein, the mutations were globally distributed on the protein (Figure 5), including in sites close to site 251, with probable local effects (Figure 5). Considering local interactions (<13 Å), we identified compensating mutations at sites 47–49, 51, 52, 212, 224, and 230. However, a general effect on protein stability could also be at play. Indeed, some local amino acids at 51 [11,12], 52 [13], and 224 [14,15] sites are also known to increase the protein’s stability (also see Supplementary text).

#### 2.4.2. Mutations with Impact on Global Stability of the Protein 

In a more global way, many compensating sites could have an effect on protein stability. Residues 51, 52, 63, 147, 153, 182, 224, and 275 are involved, or supposed to be, in the global stability of the protein [2,11,12,13,14,15,16,17,18], while the role of other residues (31, 38, 42, 175, and 184) on stabilization properties is less clear but suggested [2,13,19] (also see Supplementary Materials text).

Interestingly, this approach allowed us to display original patterns, which are described here for the first time. For example, the M182T mutation has been largely described as a high-effect compensating mutation impacting stability. Here, we highlighted other mutations at this site, such as M182C, M182F, M182I, M182L, and M182S. This diversity of compensating mutations at a remote site underlies the complexity of compensation and the importance of such comprehensive and quantitative approaches.

#### 2.4.3. Recovery Mutations in TEM-1 and Consensus Sequences of Beta-Lactamases

We then looked at the residues for which the amino acid found in TEM-1 is an exception compared to the other beta-lactamases. We found five residues: V31, N52, E58, H153, and M182. Interestingly, within these five residues, four were selected (80%) in our rescue study after selection with antibiotics (31, 52, 153, and 182). Concerning residue 31, five substitutions were selected (A, Q, M, R, and K). All of these amino acids were frequent at site 31 in the alignment of 157 beta-lactamases we used, except for M31. Concerning residues 153 and 182, which are particularly conserved within beta-lactamases, we selected some extremely frequent substitutions in the alignment of beta-lactamases (notably H153R, M182T, and M182S). This suggests that rescue possibilities are in the direction of beta-lactamase’s consensus, and that the evolution toward TEM-1 is the source of the epistasis pattern observed.

#### 2.4.4. Steps of Entrenchment of the Residue G251 in TEM-1

**Figure 6:**
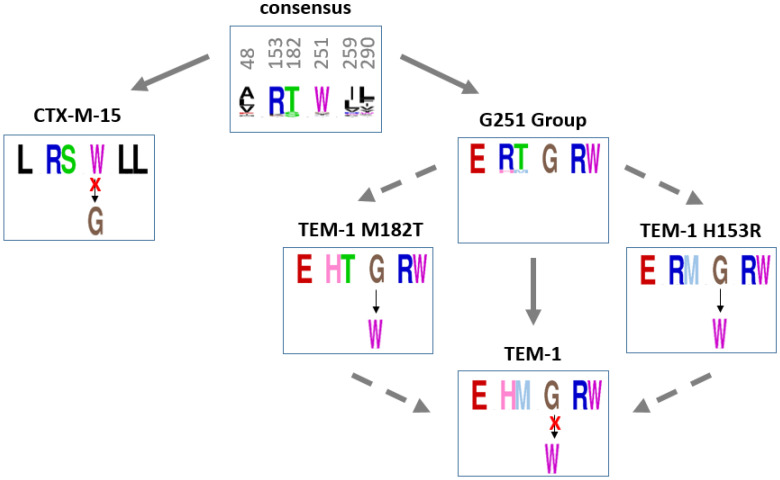
Steps of entrenchment of the residue G251 in TEM-1. The 3 sites (48, 259, and 290) that coevolve with G251 and two sites involved in compensating mutations (153 and 182) are represented beside site 251. The ability of each beta-lactamase to tolerate W251 or G251 is represented if it was validated experimentally.

On the basis of sequence alignments and of our experiments, we investigated how G251 could have been entrenched in TEM-1 (Figure 6). We focused on the sites 48, 259, and 290 (which co-evolved with G251), and on the sites 153 and 182. As mentioned above, these latter sites are of particular interest because H153 and M182 are only present in TEM-1. Furthermore, R153 and T/S182 are highly represented in class A beta-lactamases and described as stabilizing mutations in TEM-1. 

The consensus sequence obtained with the alignment of 157 beta-lactamases associates small or hydrophobic amino acids (Valine, Alanine, Leucine, or Isoleucine) in sites 48, 259, and 290; W251; and amino acids R153 and T182. 

G251 is specific to the monophyletic group of eight beta-lactamases close to TEM-1, in which it is associated with E48, R259, and W290. From a structural point of view, these sites are close to one another. We determined experimentally that G251 was incompatible with the CTX-M-15 background. Hence, our results strongly suggest an entrenchment of G251 due to coevolution with residues E48, R259, and W290. 

TEM-1 is the only beta-lactamase that harbors H153 and M182. We determined experimentally that W251 was incompatible with the TEM-1 background, but that H153R and M182T were compensatory mutations in the TEM-1 G251W variant. 

Altogether, these observations strongly suggest that TEM-1 differs from its ancestor by mutations R153H and T182M which decrease the global stability of the enzyme and later prevent a transition toward W251.

## 3. Discussion

Here, we used beta-lactamases as a model to study the context-dependency of mutation effects and proposed different steps leading to the entrenchment of a position in a protein. 

Looking at mutant libraries of TEM-1 and CTX-M-15, we found that switching residue 251 in the two backgrounds was highly deleterious in both. This observation suggests a complex evolutionary scenario to account for the evolution between these two sequences. 

Focusing on that mutation site, we then explored beta-lactamase multiple alignments. W251 was highly represented, and G251 was limited to a monophyletic group of eight beta-lactamases. In this group, G251 seems to have coevolved with three other residues. 

Then, to investigate putative compensatory mutations, we performed large libraries of mutants of TEM-1 G251W. We identified a few mutations potentially involved in local interactions and others that were not close to the residue 251 in the 3D structure but were associated with a stabilizing effect on TEM-1. The presence of several stabilizing mutations in the wild-type background of TEM-1 and the fact that TEM-1 amino acids at these sites are not dominant (compared to the beta-lactamases alignment) suggest that the TEM-1 sequence has accumulated destabilizing mutations. Several scenarios could explain this pattern. Due to strong selection, the TEM-1 initial allele that spread through the *E. coli* population may have been sampled from a succession of unlikely events and may have, therefore, accumulated by chance from some destabilizing mutations. The presence of a destabilizing methionine at position 182 of TEM-1, a unique feature of all beta-lactamases, could result from such bottlenecks. 

It is also possible that, due to stabilizing selection acting on protein stability, there can only coexist a subset of the stabilizing mutations at a given time in every beta-lactamase [20]. The presence of any destabilizing mutation could then make those stabilizing mutations beneficial. 

Finally, the diminishing return of stabilizing mutation effects on protein stability limits the selection for increased stability and makes the maintenance of all possible stabilizing mutations in the gene impossible [21]. 

Whatever scenario, it seems that a common way to compensate the G251W mutation is to improve protein stability. 

The fact that stabilizing mutations are recruited to compensate the cost of G251W mutation suggests that it affects the protein mostly through stability. In the case of the flu virus, it has been shown that destabilizing mutations can only remain in highly stable proteins [4]. However, this scenario does not seem to be sufficient to explain the presence of a G in TEM-1, as the mutation from G to W is also deleterious. Our data suggest that the G in TEM-1 has been entrenched such that its presence is tolerated in TEM-1 but not in distant relatives. Many other changes along the protein sequence could contribute to that entrenchment, but our compensatory approach found mostly distant mutations involved in protein stability. Interestingly, none of the amino acids specifically associated with the presence of a G at position 251 were found to be involved in the compensation, despite their proximity to the residue, and their own signature of entrenchment as reversion of several of these were deleterious. This suggests that, instead of a single local interaction, diffuse interactions among multiple sites are responsible for the entrenchment of the G at position 251. 

Overall, our study reveals some complex patterns of protein evolution. While local interactions allow presumably an amino acid to be entrenched, the reversion to the native state is mostly compensated by mutations having global effects on protein stability. Epistatic interactions and protein evolution may, therefore, be driven by both local and global effects. 

## 4. Materials and Methods

### 4.1. Distribution of Mutation Effects of TEM-1

For TEM-1, our data were collected from Firnberg et al.’s previous work [20] in which the MICs of amoxicillin in 5041 single missense TEM-1 mutants were determined. 

### 4.2. Distribution of Mutation Effects of CTX-M-15

#### CTX-M-15 Library Construction

CTX-M-15 mutants were constructed by using GeneMorph II Random Mutagenesis Kit (Stratagene) to obtain an average of one mutation per gene, as previously described [16]. Briefly, the mutagenized amplicons were cloned into a modified pUC19 plasmid containing the pMB1 origin of replication from pBR322, *Nco*I and *Not*I flanking the start and stop codons of CTX-M-15 ORF, and gentamicin resistance gene (*aac*C4) at the *Xba*I site. The ligation products were transformed into ElectroMax DH10B-T1 Phage Resistant *E. coli* Competent Cells (Invitrogen, Fisher Scientific) and plated on Luria-Bertani agar supplemented with gentamicin (20 mg/L). A total of 2304 randomly picked CTX-M-15 mutants were stored into 384-well microplates and sequenced by using the Sanger method.

### 4.3. MIC Measurements

The MIC was measured by a standard agar dilution method on Mueller Hinton (MH) agar plates containing a growing concentration of amoxicillin (0, 250, 500, 1000, 2000, 4000, 8000, and 16,000 mg/L). After 18 h of incubation at 37 °C, the MIC was defined as the first concentration of amoxicillin inhibiting the growth of bacteria. 

#### MIC Score

For each mutant, MIC was computed as the median of three independent MIC measurements. The MIC score is computed as log_2_(MIC). For amino acid changes that were found several times in the library as single amino acid changes, the average MIC score was retained.

The comparison of both distributions of mutation effects allows for the identification of the site 251 as a site of interest (see results).

Construction of a library of mutations in G251W TEM variants to unravel compensating mutations.

### 4.4. Strains

*E. coli* strains used in this study: 

XL1-Blue (Agilent, Santa Clara, CA) recA1 endA1 gyrA96 thi-1 hsdR17 supE44 relA1 lac [F’ proAB lacI1qZΔM15 Tn10 (Tetr)]; 

CJ236 (new England biolabs) FΔ(HindIII)::cat (Tra+ Pil+ CamR)/ ung-1 relA1 dut-1 thi-1 spoT1 mcrA; 

DH5α (Invitrogen) F– Φ80lacZΔM15 Δ(lacZYA-argF) U169 recA1 endA1 hsdR17 (rK–, mK+) phoA supE44 λ– thi-1 gyrA96 relA1.

### 4.5. Plasmids

Phagemid pSkunk3-TEM-1 was obtained graciously from Elad Firnberg and Marc Ostermeier. Phagemids pSkunk3-TEM-G251W (containing the G251W) was created by mutagenesis from pSkunk3-TEM-1 by single-step Pfunkel mutagenesis [22]. (Primers used are listed in Supplementary Table S3).

### 4.6. Pfunkel Mutagenesis

#### 4.6.1. ssDNA Production

Uracil-containing single-strand DNA of pSkunk3-TEM-G251W was produced as published by Firnberg et al. [8] and Kowalsky et al. [23] (except for the final centrifugation step, which was performed at 26,200 × G for 1 h at 4 °C). DNA was quantified by using the Qubit® ssDNA Assay Kit (ThermoFisher Scientific, Illkirch-Graffenstaden, France).

#### 4.6.2. Single Step Pfunkel Mutagenesis

Mutagenesis was performed as previously described, with 1 µg of ssDNA used as the matrix in a total volume of 100 µL. The only difference is the elongation step, which was 15 min. Thus, the reaction cycling conditions were 95 °C for 3 min, followed by 55 °C for 90 s, 68 °C for 15 min, and 45 °C for 15 min [23]. We used the innuPREP PCRpure Kit (analytik jena) to purify DNA and eluted in 15 µL of distilled DNAse/RNAse free water. A total of 2 µL was electroporated in 20 µL of DH5alpha electrocompetent cells and then incubated with 500 µL of LB media for 1 hour at 37 °C, with shaking at 250 rpm. The transformation was plated on LB agar with 50 µg/mL streptomycin and then incubated overnight at 37 °C. 

PCR verification and Sanger sequencing were performed on isolated colonies, using primers TEM-pSKUNK-DIM-F and TEM-pSKUNK-DIM-R (Supplementary Table S3), and mutants were stocked in LB-glycerol 40% after an overnight culture at 37 °C in LB media containing 50 µg/mL streptomycin. 

#### 4.6.3. Comprehensive Mutagenesis Pfunkel

Primers containing an NNS degenerated codon (N is either A, T, G, or C; S is either G or C) were designed by using the PfunkelMatlab algorithm for comprehensive codon mutagenesis, as detailed in Firnberg et al. [8] (Supplementary Table S3). One oligo is designed for each codon position replacing the codon by NNS. TEM gene was virtually tiled in 4 equal parts of 70–80 AA. Mutagenic primers were pooled according to this division. A separate Pfunkel reaction was performed for each region. Protocols were performed as published by Kowalsky et al. [23] and Firnberg et al. [8]. Purification was carried out by using the innuPREP PCR pure Kit (analytik jena), and DNA was eluted in 15 µL of distilled water. Then 2 µL was electroporated in 20 µL of DH5alpha electrocompetent cells and then incubated with 500 µL of LB media for 1 hour at 37 °C, with shaking, at 250 rpm. The transformation was plated on LB agar, with 50 µg/mL streptomycin, and then incubated overnight at 37 °C. A pool of about 50,000 colonies per tile was scrapped from LB agar plates (245 mm × 245 mm, Greiner bio-one) in LB broth and frozen at −80 °C in LB/glycerol 40%. After pooling all colonies together, plasmids were extracted from aliquot, using plasmid miniprep (Qiagen, Valencia, CA, USA). 

### 4.7. Selection Experiments

TEM G251W: For each region, 1 mL of the frozen libraries cells stocks was cultured in MH broth at 37 °C, with shaking at 250 rpm to OD_600_ 0.4, without antibiotics (named T0). Then 3.2 mL of this first culture was used to re-inoculate 96.8 mL of MH broth supplemented with 0.25 g/L of amoxicillin until OD_600_ 0.2. Half of these cultures were pelleted for freezing at −80 °C, and half were washed two consecutive times with sterile physiological serum and grown overnight in fresh MH medium, without antibiotics. Finally, half of this overnight culture was used for plasmid extraction and half was pelleted and re-suspended in LB-glycerol 40% for freezing at −80 °C. 

### 4.8. Library Preparation and Deep Sequencing

Deep sequencing was used to obtain count data of each variant in the population after selection with amoxicillin on a MiSeq, using V3 kit 2 × 300 paired-end (Illumina Technology, San Diego, CA, USA). A two-step PCR method was used to amplify independently the corresponding part of the gene and to add the Illumina sequencing adaptor and barcode sequences. In detail, plasmid DNA concentration was determined by using qubit fluorometric quantification (ThermoFisher scientific) and normalized to 2.5 ng/µL. Then 12.5 ng of DNA was used for the 1st PCR, using specific primers (depending on the tile of the gene), and allowed the attachment of an adaptor that is necessary for the 2nd PCR. Between the specific primer and the adaptor, a variable number of degenerated nucleotides (0, 1, 2, or 3 for TEM primers) were inserted in order to increase the diversity of DNA during the sequencing part on the MiSeq and to improve crosstalk and phasing calculations (Supplementary Table S3). 

Kapa Hifi Hotstart Ready Mix PCR Kit polymerase (Kapa Biosystems, Wilmington, MA, USA) was used for amplifications. The reaction cycling conditions were 95 °C for 30 s, followed by 12 cycles of 95 °C for 10 s, 55 °C for 30 s, 68 °C for 30 s, and a final extension at 68 °C for 5 min. 

After gel purification using a Qiagen gel extraction kit (Valencia, CA, USA), DNA was quantified by using Qubit Fluorometric quantification, and DNA concentration was normalized. The 2nd PCR was performed by using 5 ng of DNA, using primers commercialized by Illumina in the Nextera Index Kit, allowing the dual indexing and the addition of the remainder of the sequencing primer annealing site, along with the annealing site for the Illumina flow cell. The reaction cycling conditions were the same as previously, but only 11 cycles were performed by using a Kapa Hifi Hotstart Ready Mix PCR Kit polymerase (Kapa Biosystems). 

After gel purification with a Qiagen gel extraction kit (Valencia, CA), quantification using qPCR kappa Hifi Hotstart (Kapa Biosystems) on a Light Cycler 480 Roche was performed with reaction cycling conditions of 95 °C for 5 min, followed by 35 cycles of 95 °C for 30 s, and 60 °C for 45 s, as specified by Kapa Biosystems. All libraries corresponding to different regions of the amoxicillin concentration selection were finally pooled in equal quantity, and 12 pM was loaded on the MiSeq with a mix of 10% PhiX DNA (PhiX Control v3, Illumina) as sequencing control.

Reads were aligned on the gene sequence by using bwa [24]. 

We subsequently used the alignment of paired-end reads on the gene sequence to filter (1) mutations with conflicting sequences on the forward and reverse reads, (2) reads with insertions and deletions, and (3) reads with multiple mutations. The remaining pairs of reads (with one mutated codon or none) were then organized according to the amino acid changes they created in the sequence. We therefore created a table of counts for each possible amino acid change in the protein. Each mutation frequency was then computed, based on that of in the wild-type sequences, and translated into an enrichment factor, using the following formula: “Enrichment_Mutant_i=Mutant_i_Count(1)/Mutant_i_Count(0)*WildType(1)/WildType(0)”. 

Amino acids represented by multiple codons in the library were used as internal controls of the noise in the data set. We found a correlation of 0.91 between synonymous codons. The wild-type sequence should have disappeared after the treatment; however, due to off-site compensatory mutations, most alleles could survive the treatment, as they harbored a fraction of treatment-resistant mutants. 

As the offset mutation rate was high enough to produce a reliable compensation for most mutants, the remaining alleles were considered proportional to their real impact on survival. Alleles associated with a two-fold increase in frequency were compensatory mutations.

## Data Availability

Data are available in supplementary materials.

## Supplementary Materials

The following supporting information can be downloaded at: https://www.mdpi.com/article/10.3390/antibiotics11050652/s1. Table S1: list of compensating mutations in G251W TEM variants. The colors from white to red illustrate the increase in allelic frequency after amoxicillin selection. Table S2: Information on the 3 amino acids that are found exclusively in beta-lactamases that harbor G251: amino acid, ambler numbering positions, 3D coordinates (x, y, and z, using PDB reference 1BTL), and distance to G251 in Ångströms. Table S3: List of primers used in the study [25,26,27,28,29].
