# Supplementary material for: Local and Global Protein Interactions Contribute to Residue Entrenchment in Beta-Lactamase TEM-1
Source: Antibiotics (Basel). 2022 May 13;11(5):652. doi: 10.3390/antibiotics11050652 (PMC9137480; doi:10.3390/antibiotics11050652)
Supplement: Supplementary file 1 [file antibiotics-11-00652-s001.zip › antibiotics-1656585-supplementary.pdf]

Supplementary Materials:

Details on sites and substitutions selected after antibiotic selection

V31

The amino acid of site 31 is a valine and is located in the first alpha-helix of the enzyme. The distance to site 251 is 22 Å. Accessibility to solvent is 53 (popMusic prediction). We find 5 different mutants after antibiotic selection that have restored, at least partially the hydrolytic capacities of the enzyme. Selected mutations are V31A, V31Q, V31M, V31R, and V31K with selection coefficient between 9 and 31, V31K being the most selected. To determine if these are mutations having an impact on stability, we used popMusic predictions. A negative ΔΔG (kcal/mol) makes possible to consider that they are stabilizing mutations. Predicted ΔΔG are -0.27, -0.66, -0.02, -0.41 and -0.46 respectively.

Of these, only V31A had been reported in laboratory isolates. Two other mutants of this site have also been described in laboratory isolates and are listed in the work of Salverda but none have been found in clinical isolates [2].

D38

The amino acid of site 38 is an aspartic acid that is located in the first alpha-helix of TEM-1. The distance to 251 site is 29 Å and accessibility to solvent is 57. We find 3 mutants selected: D38R, D38N, and D38K with selection coefficient of 15, 2.2 and 19 and predicted ΔΔG of -0.23, -0,02 and -0.35 kcal/mol respectively. Of note, the 2 most selected mutations are predicted to be more stable.

Of these, only D38N had been described in clinical isolates. However, this site is frequently subject to natural evolution because D38A, D38E, D38G, D38V, and D38Y have already been described among natural isolates.

Q39

It is also located in the first alpha-helix of TEM-1. The distance to site 251 is 28Å and accessibility to solvent is 57. We found only one selected mutant: Q39K. This substitution is found in multiple clinical isolates and has been shown to increase the MIC for ceftazidime and aztreonam[25]. This mutation is also found on its own in TEM-2. The predicted ΔΔG is -0.11 kcal/mol and its frequency increased 6.5 times before and after selection. Q39K results in a charge alteration of the surrounding region of the protein and that may affect β-lactamase translocation and thus the effective concentration of enzyme in the periplasmic space[2].

A42

The substitution A42G is known to play a role in cefotaxime resistance and was frequently identified in laboratory experiments[26]. This position is 29Å far from the 251 site and accessibility is 13, which means that the site is buried. The selection coefficient was 2.5, which is quite low compared to other sites. It is not predicted to be stabilizing as ΔΔG is positive (1.23 kcal/mol). The fact that it is found in a compensatory mutation suggests that it may have an influence on the overall stability of the protein but this could be limited explaining the low selection coefficient found.

I47V, E48M, and L49M

These 3 sites are in beta-sheet number 2 of TEM-1. Their accessibility is very low (around 0) and their distance to 251 site is between 12 and 15.5 Å. Selection coefficients are 9.5, 3.5, and 30 respectively. The prediction of stability for these sites is 1.29, -0.56, and 1.15 kcal/mol respectively. Only E48M is predicted to increase stability in TEM-1. I47V has already been described in laboratory isolates and L49M has been scarcely found in clinical isolates[2]. On the opposite, E48 has no described mutants.

L51

We found 2 substitutions on L51: L51M and L51T. This site is near the 251 site (10 Å) and accessibility is low (8.5). They were selected 3.5 and 8.2 times respectively and their ΔΔG prediction is of 1.29 and 2.10 kcal/mol respectively which means that they are not supposed to be stabilizing mutations. Furthermore, all substitutions on this site have predicted stability greater than 0. This is not consistent

with the fact that 3 substitutions on this site L51I, L51F, and L51P are found in clinical isolates and that these substitutions play a role both in the activity (increase activity towards cephalosporins) and the stability of the enzyme [11,12]. It is probably its association with other mutations that increase the stability of the protein.

N52

Only N52E was selected in our experiment (selection of 3.5 times) whereas N52H, N52K, N52D, N52D, N52T, and N52Y have been found in multiple laboratory isolates. Accessibility to solvent is very high (88.5) and distance to 251 is small (12 Å). Prediction stability is -0.46 suggesting a role in stability as it is corroborated by experimental studies [13,14].

F60 and P62

F60 is in the beta-sheet number 1 of TEM and P62 is not involved in a secondary structure. Their distance to site 251 is between 21 and 22 Å and their accessibility to solvent is 20 and 25 respectively. Substitutions F60C, F60Y, F60W, P62G, and P62A were selected at respectively 2.1, 14.5, 17.5, 5.6, and 6.9 times and their prediction of stability is positive. None of these substitutions have been described elsewhere suggesting a particular interaction with site 251.

E63

Five different substitutions were selected in our experiment: E63N, E63W, E63F, E63H and E63Y with selection coefficient of 5.5, 7, 8.5, 8.5, and 32 respectively. Accessibility to solvent is high (78.5) and this site is distant to 251 (25 Å). All substitutions on site 63 are predicted to increase the stability of the protein. There is no known function for this site but several amino acid substitutions have been found at a high frequency in laboratory isolates [2].

S124

Both S124E and S124D were selected with an increased frequency of more than 5 times. This site is in the alpha-helix 4 and is located at 23Å to 251 site. Accessibility is quite high (32.5). These substitutions are not predicted to increase stability. They haven't been found in isolates elsewhere but S124G, S124N, and S124R have been found in clinical isolates and are to modulate the resistance phenotype [2].

E147

Amino acid substitutions E147A, E147G, and E147K have been found in laboratory isolates in several studies. We found only E147G in our selection experiment with an increase in the frequency of 4.6 times. Distance to 251 is of 33 Å, accessibility to solvent of 54, and prediction of ΔΔG is 1.41 kcal/mol. This substitution even if distant to the active site is probably a compensatory mutation because it has been found in compensation in several destabilized mutants laboratory[15].

H153

The substitution H153R has been found in our selection experiment (frequency has increased 10 times) in multiple clinical isolates such as other substitutions on the same site (H153D, H153L, H153Q, H153S, and H153Y). It is located in alpha-helix 6 at 30 Å to site 251 with an accessibility of 49 which is quite high. The frequent identification of this substitution in laboratory experiments suggests adaptive effects. It has been demonstrated to increase cefotaxime resistance to enzyme stability [14,15].

N175

N175 is located in the omega loop of TEM-1 at 35 Å to 251 and with very high accessibility to solvent (of 100). The substitution N175G was found 6.5 times more frequently after selection than before suggesting compensation of the destabilized site 251. An increase in protein stability is predicted (ΔΔG is -0.81 kcal/mol). Other substitutions on the same site have already been described in clinical and laboratory isolates (N175D, N175I, N175S, and N175Y) with impact on cefotaxime and ceftazidime resistance. Its localization in the omega-loop allows modifying the hydrolysis spectrum of beta-lactams [2,19,27] .

M182

M182T is the most well-known substitution in TEM backgrounds. It increases the thermodynamic stability of the enzyme and acts as a global suppressor of missense mutations that reduce enzyme stability [15–18]. Another explanation for the mode of action of M182T could be that it suppresses aggregation and misfolding induced by other mutations [28]. We found this substitution with an increase in the frequency of 340 times making her the most compensatory mutation. However, we also found other substitutions at variable frequencies such as M182S, M182C, M182F, M182I, M182L, and M182A with selection coefficient of 144, 14, 10, 2.5, and 2.3 respectively. The large compensation with the M182S mutation is also remarkable and has not yet been described in natural isolates. This site is located at 23.2 Å to site 251 and has medium accessibility of 19. It is not predicted to be stabilizing regarding the popMusic prediction.

A184

Site 184 is at 20 Å to site 251 and has low accessibility of 12. Three substitutions A184V, A184P, and A184L have been selected at frequencies of 3.5, 2.8, and 66 times in our experiments showing a strong compensation of this site. No increase in stability is predicted. Two other substitutions A184T and A184V have been found in laboratory isolates but not in our experiment.

E212

We observe a strong selection on site 212 which is located near site 251 (12.5Å) with accessibility of 46. It is in the alpha-helix 9. This site has never been described as being of particular interest on stability, which is corroborated by prediction stability ($\Delta\Delta G>0$). Its proximity to site 251 suggests local epistasis and particularly with the substitution E212A which is strongly selected (X46.5) but also with E212M and E212S (both frequencies were multiplied by 7).

G218

The substitution G218D was selected 3.5 times after the selection experiment. It is located at 17.3 Å of site 251 and is not implicated in a particular secondary structure. Prediction stability is 0.05 kcal/mol and thus, the mutation seems to be quite neutral. Only the substitution G218E has been described in clinical isolates with no known effect.

A224

This site is located in the alpha-helix 10 near site 251, at 12.6 Å with accessibility of 22. The substitution A224V was strongly selected with a selection coefficient of 34.5. This substitution found in clinical isolates is known to increase enzyme stability [14,15]. It is also located in proximity to site 251 which makes possible a local interaction in our protein.

F230

Located in beta-sheet number 5 of TEM, F230 is one of the closest amino acids of site 251 (located at 5.5 Å) with small accessibility to solvent (of 11). The selection of the substitution F230K (X 5.4 times) is very probably due to a very strong local epistasis. No increase in stability is predicted using popMusic and this site had never been described as playing a role in the stability of phenotype of resistance.

R275

The R275 is located in the 12th alpha-helix of TEM-1 with very low accessibility to solvent and at 24 Å to site 251. It is known to be involved in increasing the resistance to inactivation by clavulanate, a beta-lactamase inhibitor, but also in the resistance of cefotaxime. Furthermore, its effect on stability has been well documented and particularly for substitutions R275L and R275Q that were both found in studies selecting for stabilizing mutations [12,15,29]. Substitutions at residue 275 may be assimilated to a global suppressor mutation[17]. We found 3 substitutions selected: R275I was strongly selected with a selection coefficient of 54 but also R275T and R275V which were selected about 2.5 times. Only R275I has a negative $\Delta\Delta G$ (-0.14 kcal/mol) corroborating its potential impact on stability.

Table **S1.** list of compensating mutations in G251W TEM variants. The colors from white to red illustrate the increase in allelic frequency after amoxicillin selection.

Selected mutation (TEM-1 G251W)	Increase allele frequency under amoxicillin selection
V31A	8.91
V31K	30.75
V31M	16.81
V31Q	11.06
V31R	17.21
D38K	18.91
D38N	2.22
D38R	15.03
Q39K	6.25
A42G	2.28
I47V	9.56
E48M	3.55
L49M	30.19
L51M	3.66
L51T	8.24
N52E	3.35
F60C	2.10
F60W	17.37
F60Y	14.53
P62A	16.91
P62G	9.90
E63F	8.44
E63H	8.46
E63N	5.60
E63W	6.85
E63Y	32.10
S124E	5.18
S124D	5.30
E147G	4.61
H153R	10.14
N175G	6.61
M182A	2.31
M182C	13.99
M182F	10.19

M182I	7.07
M182L	2.47
M182S	143.79
M182T	339.62
A184L	66.24
A184P	2.85
A184V	3.62
E212A	46.59
E212M	7.03
E212S	7.09
G218D	3.16
A224A	2.93
A224V	34.64
F230K	5.39
R275I	53.76
R275T	2.61
R275V	2.28

Increase in allele frequency	1	5	10	20	30	50	75	100

Table S2. Informations on the 3 amino acids that are found exclusively in beta-lactamases that harbor G251: amino acid, ambler numbering positions, 3D coordinates (x, y and z using PDB reference 1BTL) and distance to G251 in Ångströms.

Amino acid	Ambler position	pos_x (Calpha)	pos_y (Calpha)	pos_z (Calpha)	distance to 251 site (Å)
GLU	48	2.614	-0.909	18.168	12.2
GLY	251	-7.683	5.219	20.315	0.0
ARG	259	-3.89	-0.063	20.442	6.5
TRP	290	-3.544	5.42	11.889	9.4

Table S3: list of primers used in the study

oligo name	5'-3' sequence
TEM-mut-G251W	ATCATTGCAGCACTGTGGCCAGATGGTAAGCC
TEM-pSKUNK-DIM-F	CTGTTGACAATTAATCATCGG
TEM-pSKUNK-DIM-R	GAACGCCAGCAAGACGTAG
NGS-TEM-part1-1-F	5′ TCGTCGGCAGCGTCAGATGTGTATAAGAGACAGAATTTCACACAGGAGGAAGGA
NGS-TEM-part1-2-F	5′ TCGTCGGCAGCGTCAGATGTGTATAAGAGACAGNAATTTCACACAGGAGGAAGGA
NGS-TEM-part1-3-F	5′ TCGTCGGCAGCGTCAGATGTGTATAAGAGACAGNNAATTTCACACAGGAGGAAGGA
NGS-TEM-part1-4-F	5′ TCGTCGGCAGCGTCAGATGTGTATAAGAGACAGNNNAATTTCACACAGGAGGAAGGA
NGS-TEM-part1-1-R	5′ GTCTCGTGGGCTCGGAGATGTGTATAAGAGACAGAGTTGCTCTTGCCCGGCGT
NGS-TEM-part1-2-R	5′ GTCTCGTGGGCTCGGAGATGTGTATAAGAGACAGNAGTTGCTCTTGCCCGGCGT
NGS-TEM-part1-3-R	5′ GTCTCGTGGGCTCGGAGATGTGTATAAGAGACAGNNAGTTGCTCTTGCCCGGCGT
NGS-TEM-part1-4-R	5′ GTCTCGTGGGCTCGGAGATGTGTATAAGAGACAGNNNAGTTGCTCTTGCCCGGCGT
NGS-TEM-part2-1-F	5′ TCGTCGGCAGCGTCAGATGTGTATAAGAGACAGAATGATGAGCACTTTTAAAGTTCT
NGS-TEM-part2-2-F	5′ TCGTCGGCAGCGTCAGATGTGTATAAGAGACAGNAATGATGAGCACTTTTAAAGTTCT
NGS-TEM-part2-3-F	5′ TCGTCGGCAGCGTCAGATGTGTATAAGAGACAGNNAATGATGAGCACTTTTAAAGTTCT
NGS-TEM-part2-4-F	5′ TCGTCGGCAGCGTCAGATGTGTATAAGAGACAGNNNAATGATGAGCACTTTTAAAGTTCT
NGS-TEM-part2-1-R	5′ GTCTCGTGGGCTCGGAGATGTGTATAAGAGACAGAACGATCAAGGCGAGTTACAT
NGS-TEM-part2-2-R	5′ GTCTCGTGGGCTCGGAGATGTGTATAAGAGACAGNAACGATCAAGGCGAGTTACAT
NGS-TEM-part2-3-R	5′ GTCTCGTGGGCTCGGAGATGTGTATAAGAGACAGNNAACGATCAAGGCGAGTTACAT
NGS-TEM-part2-4-R	5′ GTCTCGTGGGCTCGGAGATGTGTATAAGAGACAGNNNAACGATCAAGGCGAGTTACAT
NGS-TEM-part3-1-F	5′ TCGTCGGCAGCGTCAGATGTGTATAAGAGACAGACAACGATCGGAGGACCGAA
NGS-TEM-part3-2-F	5′ TCGTCGGCAGCGTCAGATGTGTATAAGAGACAGNACAACGATCGGAGGACCGAA

NGS-TEM-part3-3-F	5′ TCGTCGGCAGCGTCAGATGTGTATAAGAGACAGNNACAACGATCGGAGGACCGAA
NGS-TEM-part3-4-F	5′ TCGTCGGCAGCGTCAGATGTGTATAAGAGACAGNNNACAACGATCGGAGGACCGAA
NGS-TEM-part3-1-R	5′ GTCTCGTGGGCTCGGAGATGTGTATAAGAGACAGTCCAGATTTATCAGCAATAAACCA
NGS-TEM-part3-2-R	5′ GTCTCGTGGGCTCGGAGATGTGTATAAGAGACAGNTCCAGATTTATCAGCAATAAACCA
NGS-TEM-part3-3-R	5′ GTCTCGTGGGCTCGGAGATGTGTATAAGAGACAGNNTCCAGATTTATCAGCAATAAACCA
NGS-TEM-part3-4-R	5′ GTCTCGTGGGCTCGGAGATGTGTATAAGAGACAGNNNTCCAGATTTATCAGCAATAAACCA
NGS-TEM-part4-1-F	5′ TCGTCGGCAGCGTCAGATGTGTATAAGAGACAGAGGCGGATAAAGTTGCAGGA
NGS-TEM-part4-2-F	5′ TCGTCGGCAGCGTCAGATGTGTATAAGAGACAGNAGGCGGATAAAGTTGCAGGA
NGS-TEM-part4-3-F	5′ TCGTCGGCAGCGTCAGATGTGTATAAGAGACAGNNAGGCGGATAAAGTTGCAGGA
NGS-TEM-part4-4-F	5′ TCGTCGGCAGCGTCAGATGTGTATAAGAGACAGNNNAGGCGGATAAAGTTGCAGGA
NGS-TEM-part4-1-R	5′ GTCTCGTGGGCTCGGAGATGTGTATAAGAGACAGCGGTCGAATGCATAAGCTTACT
NGS-TEM-part4-2-R	5′ GTCTCGTGGGCTCGGAGATGTGTATAAGAGACAGNCGGTCGAATGCATAAGCTTACT
NGS-TEM-part4-3-R	5′ GTCTCGTGGGCTCGGAGATGTGTATAAGAGACAGNNCGGTCGAATGCATAAGCTTACT
NGS-TEM-part4-4-R	5′ GTCTCGTGGGCTCGGAGATGTGTATAAGAGACAGNNNCGGTCGAATGCATAAGCTTACT

1. Hall, B.G.; Barlow, M. Evolution of the Serine Beta-Lactamases: Past, Present and Future. *Drug Resist. Updates* **2004**, *7*, 111–123. https://doi.org/10.1016/j.drup.2004.02.003.
2. Salverda, M.L.M.; De Visser, J.A.G.M.; Barlow, M. Natural Evolution of TEM-1 β-Lactamase: Experimental Reconstruction and Clinical Relevance. *FEMS Microbiol. Rev.* **2010**, *34*, 1015–1036. https://doi.org/10.1111/j.1574-6976.2010.00222.x.
3. Figliuzzi, M.; Jacquier, H.; Schug, A.; Tenaillon, O.; Weigt, M. Coevolutionary Landscape Inference and the Context-Dependence of Mutations in Beta-Lactamase TEM-1. *Mol. Biol. Evol.* **2016**, *33*, 268–280. https://doi.org/10.1093/molbev/msv211.
4. Gong, L.I.; Suchard, M.A.; Bloom, J.D. Stability-Mediated Epistasis Constrains the Evolution of an Influenza Protein. *Elife* **2013**, *2*, e00631. https://doi.org/10.7554/eLife.00631.
5. Kondrashov, A.S.; Sunyaev, S.; Kondrashov, F.A. Dobzhansky-Muller Incompatibilities in Protein Evolution. *Proc. Natl. Acad. Sci. USA* **2002**, *99*, 14878–14883. https://doi.org/10.1073/pnas.232565499.
6. Jordan, D.M.; Frangakis, S.G.; Golzio, C.; Cassa, C.A.; Kurtzberg, J.; Task Force for Neonatal Genomics; Davis, E.E.; Sunyaev, S.R.; Katsanis, N. Identification of Cis-Suppression of Human Disease Mutations by Comparative Genomics. *Nature* **2015**, *524*, 225–229. https://doi.org/10.1038/nature14497.
7. Shah, P.; McCandlish, D.M.; Plotkin, J.B. Contingency and Entrenchment in Protein Evolution under Purifying Selection. *Proc. Natl. Acad. Sci. USA* **2015**, *112*, E3226–E3235. https://doi.org/10.1073/pnas.1412933112.
8. Firnberg, E.; Labonte, J.W.; Gray, J.J.; Ostermeier, M. A Comprehensive, High-Resolution Map of a Gene's Fitness Landscape. *Mol. Biol. Evol.* **2014**, *31*, 1581–1592. https://doi.org/10.1093/molbev/msu081.
9. Marciano, D.C.; Brown, N.G.; Palzkill, T. Analysis of the Plasticity of Location of the Arg244 Positive Charge within the Active Site of the TEM-1 Beta-Lactamase. *Protein Sci.* **2009**, *18*, 2080–2089. https://doi.org/10.1002/pro.220.
10. Naas, T.; Oueslati, S.; Bonnin, R.A.; Dabos, M.L.; Zavala, A.; Dortet, L.; Retailleau, P.; Iorga, B.I. Beta-Lactamase Database (BLDB)—Structure and Function. *J. Enzym. Inhib. Med. Chem.* **2017**, *32*, 917–919. https://doi.org/10.1080/14756366.2017.1344235.
11. Caporale, B.; Franceschini, N.; Perilli, M.; Segatore, B.; Rossolini, G.M.; Amicosante, G. Biochemical Characterization of Laboratory Mutants of Extended-Spectrum Beta-Lactamase TEM-60. *Antimicrob. Agents Chemother.* **2004**, *48*, 3579–3582. https://doi.org/10.1128/AAC.48.9.3579-3582.2004.
12. Osuna, J.; Pérez-Blancas, A.; Soberón, X. Improving a Circularly Permuted TEM-1 Beta-Lactamase by Directed Evolution. *Protein Eng.* **2002**, *15*, 463–470. https://doi.org/10.1093/protein/15.6.463.
13. Hecky, J.; Müller, K.M. Structural Perturbation and Compensation by Directed Evolution at Physiological Temperature Leads to Thermostabilization of Beta-Lactamase. *Biochemistry* **2005**, *44*, 12640–12654. https://doi.org/10.1021/bi0501885.
14. Bershtein, S.; Goldin, K.; Tawfik, D.S. Intense Neutral Drifts Yield Robust and Evolvable Consensus Proteins. *J. Mol. Biol.* **2008**, *379*, 1029–1044. https://doi.org/10.1016/j.jmb.2008.04.024.
15. Kather, I.; Jakob, R.P.; Dobbek, H.; Schmid, F.X. Increased Folding Stability of TEM-1 Beta-Lactamase by in Vitro Selection. *J. Mol. Biol.* **2008**, *383*, 238–251. https://doi.org/10.1016/j.jmb.2008.07.082.
16. Jacquier, H.; Birgy, A.; Le Nagard, H.; Mechulam, Y.; Schmitt, E.; Glodt, J.; Bercot, B.; Petit, E.; Poulain, J.; Barnaud, G.; et al. Capturing the Mutational Landscape of the Beta-Lactamase TEM-1. *Proc. Natl. Acad. Sci. USA* **2013**, *110*, 13067–13072. https://doi.org/10.1073/pnas.1215206110.
17. Huang, W.; Palzkill, T. A Natural Polymorphism in Beta-Lactamase Is a Global Suppressor. *Proc. Natl. Acad. Sci. USA* **1997**, *94*, 8801–8806. https://doi.org/10.1073/pnas.94.16.8801.
18. Bloom, J.D.; Silberg, J.J.; Wilke, C.O.; Drummond, D.A.; Adami, C.; Arnold, F.H. Thermodynamic Prediction of Protein Neutrality. *Proc. Natl. Acad. Sci. USA* **2005**, *102*, 606–611. https://doi.org/10.1073/pnas.0406744102.
19. Barlow, M.; Hall, B.G. Predicting Evolutionary Potential: In Vitro Evolution Accurately Reproduces Natural Evolution of the Tem Beta-Lactamase. *Genetics* **2002**, *160*, 823–832. https://doi.org/10.1093/genetics/160.3.823.
20. DePristo, M.A.; Weinreich, D.M.; Hartl, D.L. Missense Meanderings in Sequence Space: A Biophysical View of Protein Evolution. *Nat. Rev. Genet.* **2005**, *6*, 678–687. https://doi.org/10.1038/nrg1672.
21. Wylie, C.S.; Shakhnovich, E.I. A Biophysical Protein Folding Model Accounts for Most Mutational Fitness Effects in Viruses. *Proc. Natl. Acad. Sci. USA* **2011**, *108*, 9916–9921. https://doi.org/10.1073/pnas.1017572108.
22. Firnberg, E.; Ostermeier, M. PFunkel: Efficient, Expansive, User-Defined Mutagenesis. *PLoS ONE* **2012**, *7*, e52031. https://doi.org/10.1371/journal.pone.0052031.
23. Kowalsky, C.A.; Klesmith, J.R.; Stapleton, J.A.; Kelly, V.; Reichkitzer, N.; Whitehead, T.A. High-Resolution Sequence-Function Mapping of Full-Length Proteins. *PLoS ONE* **2015**, *10*, e0118193. https://doi.org/10.1371/journal.pone.0118193.
24. Li, H.; Durbin, R. Fast and Accurate Long-Read Alignment with Burrows-Wheeler Transform. *Bioinformatics* **2010**, *26*, 589–595. https://doi.org/10.1093/bioinformatics/btp698.

25. Blazquez, J.; Morosini, M.I.; Negri, M.C.; Gonzalez-Leiza, M.; Baquero, F. Single Amino Acid Replacements at Positions Altered in Naturally Occurring Extended-Spectrum TEM Beta-Lactamases. *Antimicrob. Agents Chemother.* **1995**, *39*, 145–149. https://doi.org/10.1128/AAC.39.1.145.
26. Stemmer, W.P. Rapid Evolution of a Protein in Vitro by DNA Shuffling. *Nature* **1994**, *370*, 389–391. https://doi.org/10.1038/370389a0.
27. Ortlund, E.A.; Bridgham, J.T.; Redinbo, M.R.; Thornton, J.W. Crystal Structure of an Ancient Protein: Evolution by Conformational Epistasis. *Science* **2007**, *317*, 1544–1548. https://doi.org/10.1126/science.1142819.
28. Sideraki, V.; Huang, W.; Palzkill, T.; Gilbert, H.F. A Secondary Drug Resistance Mutation of TEM-1 Beta-Lactamase That Suppresses Misfolding and Aggregation. *Proc. Natl. Acad. Sci. USA* **2001**, *98*, 283–288. https://doi.org/10.1073/pnas.98.1.283.
29. Chaïbi, E.B.; Sirot, D.; Paul, G.; Labia, R. Inhibitor-Resistant TEM Beta-Lactamases: Phenotypic, Genetic and Biochemical Characteristics. *J. Antimicrob. Chemother.* **1999**, *43*, 447–458. https://doi.org/10.1093/jac/43.4.447.
